# Peripheral Auditory Pathway and ABR Characterization in Adults with Williams Syndrome *

**DOI:** 10.1055/s-0044-1785457

**Published:** 2024-07-05

**Authors:** Jacqueline Aquino do Nascimento, Liliane Aparecida Fagundes Silva, Alessandra Gianella Samelli, Carla Gentile Matas

**Affiliations:** 1 Department of Physical, Speech-Language-Hearing, and Occupational Therapies, School of Medicine, Universidade de São Paulo (FMUSP), São Paulo, SP, Brazil.

**Keywords:** Williams syndrome, hearing, hearing loss

## Abstract

**Introduction** Williams syndrome (WS) is a genetic disorder caused by a microdeletion in chromosome 7, affecting ∼ 28 genes. Studies have demonstrated conductive losses seemingly related to the absence of the *elastin* gene and mild to profound sensorineural losses due to cochlear fragility.

**Objective** To characterize and compare the peripheral auditory system and auditory brainstem response (ABR) of adults with WS and neurotypical adults matched by age and gender.

**Methods** We conducted a cross-sectional observational study with 30 individuals of both sexes, aged 18 to 37 years – 15 of them with WS (study group) and 15 with neither the syndrome nor hearing complaints (control group), matched for sex and age. The subjects underwent pure-tone and speech audiometry, acoustic immittance, transient-evoked otoacoustic emissions (TEOAEs), and ABR.

**Results** Early-onset sensorineural hearing loss was found in 53.3% of the study sample, mostly mild, occurring above 3 kHz. The TEOAEs were absent in 53.3% of assessed subjects; for those in whom they were present, the signal-to-noise responses were significantly lower than in the control group. In the ABR, increased absolute latencies were observed in waves I and III.

**Conclusion** Individuals with WS have early and progressive cochlear impairments, mainly affecting the basal region of the cochlea. They may have low brainstem changes which seem to begin in adulthood.

## Introduction

Williams syndrome (WS) is a genetic disorder caused by a microdeletion in the chromosomal region 7q11.23, affecting ∼ 28 genes 1 2

Findings in the literature point to a range of possible hearing impairments in WS, such as conductive and sensorineural hearing loss, greater and progressive impairments at higher frequencies, hyperacusis, and subclinical signs of hearing changes, demonstrated by the absence and/or lower amplitude of otoacoustic emissions (OAEs). 3 4 5 6 7 8 9 10 11 12 13 14 15

Auditory changes in subjects with WS may be due to *elastin* ( *ELN* ) gene deficiency, leading to abnormalities in middle ear structures, such as an increase in pressure of the tympanic membrane and the tendon of the stapedius muscle, as well as characteristic abnormalities of the Eustachian tube that may impair pressure maintenance within the ears and thus sound transmission. 15 The absence of this gene is also related to lack of cochlear perfusion, which would lead to a decrease in outer hair cells, thus justifying sensorineural hearing loss. 6

Also, according to the literature, the lack of other genes like *LIMK1* and *GTF2IRD1* may also be related to the auditory phenotype of these subjects. 14 16

Regarding central auditory pathways, only two studies have applied electrophysiological tests to assess hearing in WS patients. Moreover, the results of extant studies do not agree with one another. 11 17

A study 17 reported longer latencies of waves I, III, and V with normal interpeak latencies in individuals with WS compared with typically developing individuals. According to the authors, 17 this result suggests that the increased latencies of waves III and V were related to a delay in wave I latency, indicating desynchronization of auditory nerve fibers or dysfunction in the interaction between cochlear hair cells and the distal portion of the auditory nerve. However, this study included the evaluation of patients with external acoustic meatus abnormalities (95% of the cases), type B tympanometric curve (19% of the cases), and conductive hearing loss detected by pure tone audiometry (10% of the cases); the participants with typical development did not present any of these abnormalities.

In 2008, another study 18 suggested that the results of the aforementioned study 17 could indicate cochlear nerve damage, which leads to dysfunction in the synchronous activity of the afferent auditory nerve pathway in WS. However, further studies are required to confirm this hypothesis.

To clarify this hypothesis, in 2012 a study 10 evaluated 14 individuals with WS, with no middle ear impairment, and reported normal ABR in all subjects. These results rule out retrocochlear impairment for the WS population; however, the absence of a control group with typical development limits the comparison and confirmation of these findings.

Therefore, further evidence of the function of both peripheral and central auditory pathways in WS individuals, including a systematically controlled comparison group, may fill current gaps in the pathophysiology of hearing impairment in WS, helping to improve the quality of life in this population.

## Objective

To characterize and compare the peripheral auditory system and ABR results of adults with WS and neurotypical adults matched by age and gender.

## Methods

The present is a cross-sectional observational study of individuals with WS. The project was approved (under process no. 2.504.522) by the Research Ethics Committee of the institution where the study was developed.

### Participants

The study sample comprised 30 individuals of both sexes with chronological ages ranging from 18 to 37 years. The study group (SG) was composed of 15 subjects with WS, with the exclusion of individuals with difficulties understanding commands to perform the tests, excessive cerumen in the external acoustic meatus, and type-B tympanogram. The control group (CG) was composed of 15 neurotypical individuals with no hearing complaints, matched for sex and age with SG individuals. The inclusion criterion was the absence of hearing and neurological impairments.

### Audiological Assessment

First, the medical history of the subjects was surveyed, and their external acoustic meatus was also inspected.

### Acoustic Immittance

Tympanometry and ipsilateral and contralateral acoustic reflexes were evaluated at 0.5 kHz, 1 kHz, 2 kHz, and 4 kHz to identify possible middle ear impairments. The tympanometric curve was classified according to Jerger (1970).

### Pure-tone Threshold Audiometry

Hearing thresholds were determined with pure-tone audiometry at 250 Hz to 8,000 Hz, using supra-aural earphones (model TDH-39, Telephonics, Farmingdale, NY, United States), and speech audiometry. When air-conduction (AC) thresholds were higher than 25 dB, bone-conduction (BC) thresholds were assessed with a bone vibrator at 0.5 kHz to 4 kHz. The type of hearing loss was determined based on the following criteria: conductive hearing loss (thresholds: BC ≤ 15 dB; AC ≥ 25 dB; and air-bone gap ≥ 15 dB), mixed hearing loss (thresholds: BC > 15 dB; AC > 25 dB; and air-bone gap ≥ 15 dB), and sensorineural hearing loss (thresholds: BC > 15 dB; AC > 25 dB; and air-bone gap ≤ 10 dB). The degree of hearing loss was determined based on the 3-frequency mean, with low and medium (0.5 kHz, 1 kHz, and 2 kHz) 19 and high frequencies (3 kHz, 4 kHz, and 6 kHz), as follows: 26 dB to 40 dB – mild; 41 dB to 55 dB – moderate; 56 dB to 70 dB – moderately severe; 71 dB to 90 dB – severe; and ≥ 91 dB – profound. 20 In the case of hearing losses at different frequencies, each frequency was considered separately. Subjects were considered with hearing loss when at least one ear presented an abnormality.

### Transient-evoked Otoacoustic Emissions (TEOAEs)

Transient-evoked otoacoustic emissions (TEOAEs) were obtained using 1,024 nonlinear click stimuli at 80 dB SPL, with a 30-ms analysis window, duration of 75 μs, 19.30/s presentation rate, and 4,000 gain, assessing the frequencies of 1 kHz, 1.5 kHz, 2 kHz, 3 kHz, and 4 kHz. The subjects were instructed to remain seated and still throughout the examination.

The presence of TEOAEs was verified with ≥ 70% probe stability, ≥ 50% response reproducibility, and signal-to-noise ratios (SNRs) > 3 dB SPL (at 1 kHz and 1.5 kHz) and > 6 dB SPL (at the other frequencies). The TEOAE was considered present when there had been responses in at least three frequency bands. In the case of failure, the probe was repositioned to retake measures; the best response was considered. 21

### Auditory Brainstem Response (ABR)

The subjects' skin was cleaned with an abrasive paste, and then the silver/silver chloride (Ag/AgCl) electrodes were placed with conductive paste and micropore tape to record the auditory brainstem response (ABR). The electrodes were positioned following the standards of the ten-twenty International Electrode System (IES) 22 –active electrode (Fz) and ground electrode (Fpz) positioned on the forehead, and reference electrodes, on the left (M1) and right mastoids (M2).

The ABR was picked up by presenting rarefaction polarity click stimuli at 80 dBnHL monaurally through insert earphones, at the 27.7/s presentation rate; 100-Hz high-pass and 1,500-Hz low-pass filters were used, with a 12-ms recording window. Two sweeps with 2,048 stimuli each were collected, aiming to obtain responses and reproducibility of the tracing.

The qualitative analysis was based on the absolute latencies of waves I, III, and V and interpeak intervals I-III, III-V, and I-V, following the normal criteria described in the literature.

### Statistical Analysis

Descriptive and inferential analyses were conducted using the Minitab Statistical Software (Minitab, LLC, State College, PA, United States), version 19. The inferential analysis used parametric statistical tests (when the sample followed a normal distribution) and nonparametric tests (when the sample did not follow a normal distribution or meet parametric test assumptions).

Each assessment result was compared between ears and groups. Hence, two-way analysis of variance (ANOVA) or the Kruskal-Wallis test was used for multiple comparisons. The post-hoc analysis was performed through the Fisher least significant difference (LSD) parametric test, or Mann-Whitney (to compare groups) or Wilcoxon nonparametric tests (to compare left and right ears).

The Pearson Chi-squared (χ ^2^ ) test was used to verify associations between two categorical variables, such as the presence/absence of responses or changes. A correlation analysis was also performed regarding chronological age and the mean hearing thresholds in the SG with the Pearson correlation coefficient.

The level of statistical significance in all analyses was set at *p* ≤ 0.05 (5%). 23 24

## Results

**Fig. 1:**
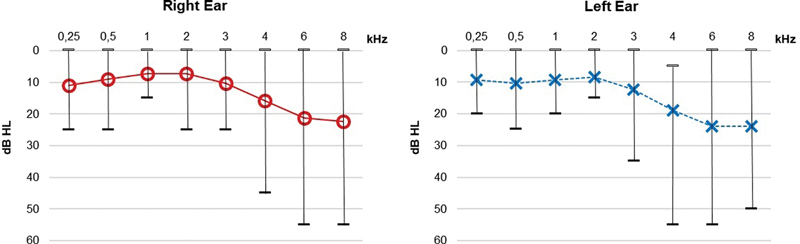
Average auditory thresholds in subjects with WS. Caption: CG – control group; SG – study group; dBHL- decibel - hearing level.

All the patients had a type-A tympanometric curve. Among the 15 patients with WS evaluated, 8 had hearing loss (53.3% of the sample), all of which were of the sensorineural type (unilateral in 2 patients and bilateral in 6 patients). The degree of hearing loss was mild to moderate, affecting frequencies above 3 kHz ( Fig. 1 ).

**Fig. 2:**
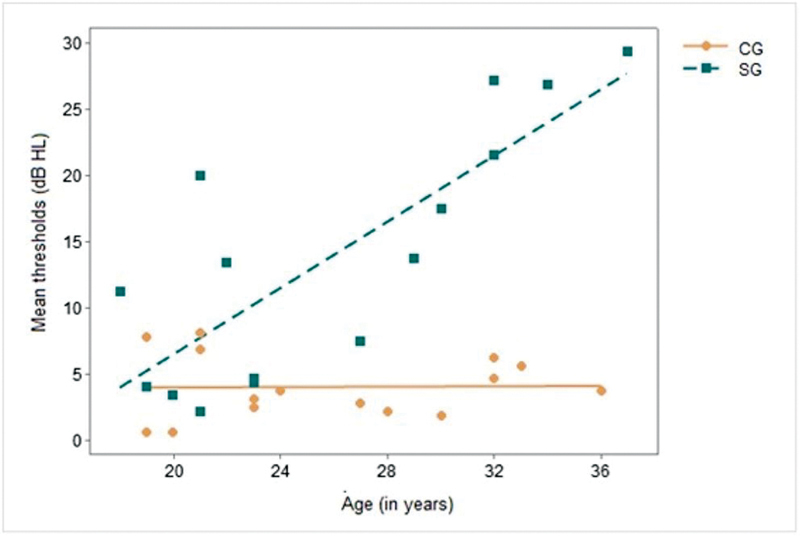
Correlation between chronological age and mean hearing thresholds. Caption: CG – control group; SG – study group; dBHL- decibel - hearing level.

The influence analysis of age on hearing loss in subjects with WS showed a positive correlation in the SG – the older the individual, the higher the hearing thresholds (r = 0.801; confidence interval [CI] = 0.490–0.931; *p* < 0.000) ( Fig. 2 ).

**Fig. 3:**
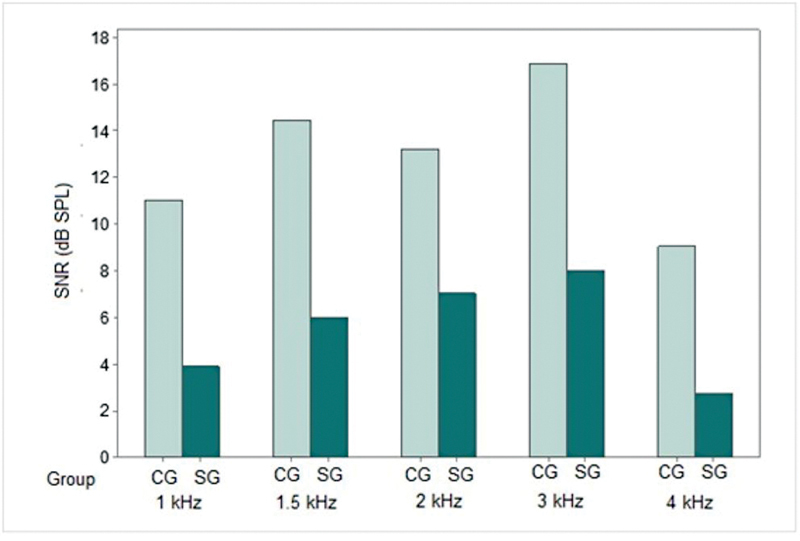
Mean TEOAE signal-to-noise ratio in both groups at each frequency. Caption: CG – control group; SG – study group; SNR – signal-to-noise ratio; dBSPL - decibel sound pressure level; kHz- kilohertz.

In the present study, we observed absence of TEOAEs in 8 SG subjects (53,3%), 6 of whom also showed hearing loss. The TEOAE analysis showed statistically significant differences between the groups: the SG presented weaker TEOAE responses than the CG at all frequencies assessed; the higher the frequency, the greater the difference between the groups ( Fig. 3 ).

In the ABR assessment, longer latencies were observed in at least one ear in all patients with WS. In general, the main abnormality was an increase in the absolute latency of waves I and III, and there was no difference between the ears ( Table 1 ). All CG individuals presented normal ABR latencies.

**Table 1:** Qualitative analysis of the auditory brainstem response (ABR; normal or abnormal) and *p* -value of the association between the variable ‘ABR response’ and the right and left ears

	Ear	Normal	Abnormal	Chi-squared	*p* -value
**Wave I**	Right	46.66%	53.33%	0.136	0.713
	Left	40.00%	60.00%		
**Wave III**	Right	26.66%	73.33%	2.222	0.136
	Left	53.33%	46.66%		
**Wave V**	Right	86.66%	13.33%	0.000	1.000
	Left	86.66%	13.33%		
**Interpeak intervals I-III**	Right	80.00%	20.00%	0.000	1.000
	Left	80.00%	20.00%		
**Interpeak intervals III-V**	Right	93.33%	6.66%	1.154	0.283
	Left	80.00%	20.00%		
**Interpeak intervals I-V**	Right	93.33%	6.66%	0.370	0.543
	Left	86.66%	13.33%		

**Fig. 4:**
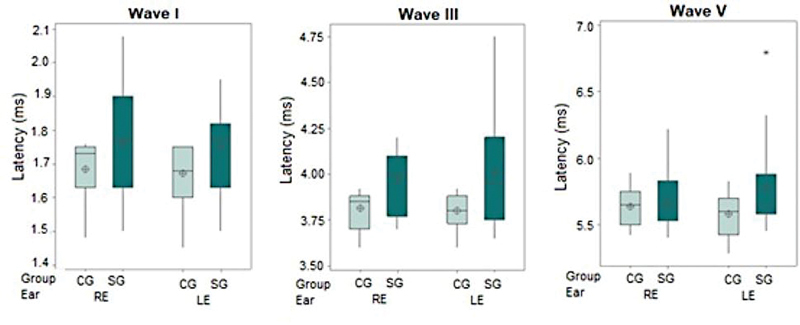
Boxplot of the absolute latencies of waves I, III, and V in BAEP per group and ear. Caption: CG – control group; SG – study group; RE- right ear; LE- left ear; ms- milliseconds.

**Fig. 5:**
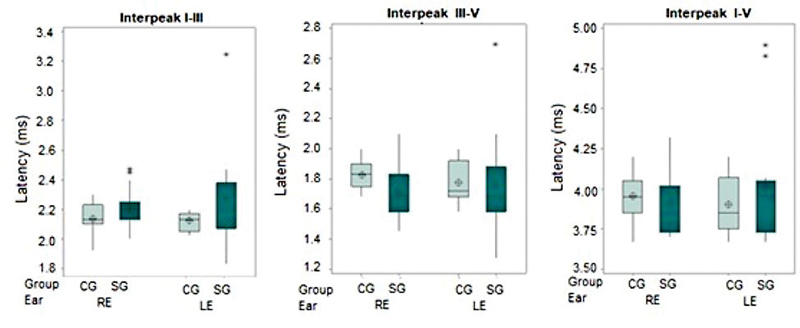
Boxplot of the latencies of interpeak intervals I-III, III-V, and I-V in BAEP per group and ear Caption: CG – control group; SG – study group; RE- right ear; LE- left ear; ms- milliseconds.

The ABR absolute latencies of waves I, III, and V ( Fig. 4 ) and the latencies of interpeak intervals I-III, III-V, and I-V ( Fig. 5 ) were analyzed. Statistically significant differences were found only in the absolute latency of wave III, although there was also a trend toward statistical significance in the absolute latency of wave I.

## Discussion

The objective of the present study was to characterize and compare the peripheral auditory system and ABR results of adults with WS and neurotypical adults matched by age and gender.

A total of 15 individuals with WS were assessed, and in 8 (53,3%) patients hearing thresholds were impaired; these patients presented sensorineural hearing loss above 3 kHz – unilateral in 2 (25%) and bilateral in 6 (75%). These results are in line with those of previous studies 4 6 7 8 9 10 11 13 that also found hearing loss or considerably increased thresholds, predominantly at higher frequencies.

The present study assessed the hearing of adults with ages ranging from 18 to 37 years, showing that increased thresholds were more consistent starting at 27 years old – after this age, all subjects increasingly had some degree of hearing loss. The positive correlation found in the present study between chronological age and hearing loss is a strong indicator that hearing loss in this population can be progressive ( Fig. 2 ). These data agree with those of another study, 6 which assessed adults and children with WS and found 100% of impairment in the adults. The data of the present study also corroborate that of another one, 4 which assessed 16 adults with WS older than 30 years of age and found hearing losses in 75% of the sample. Likewise, some authors conducted a longitudinal study 11 in children aged 5 to 14 years and verified the hearing thresholds increased by 10 dB in 10 years. This suggests that hearing losses in subjects with WS have an early onset and are progressive.

Another study 16 considers that progressive hearing loss in subjects with WS can be a consequence of reduced expression of the *LIMK1* gene in the cochlear hair cells. This gene is one of those responsible for motility homeostasis in outer hair cells; hence, disruptions in this system would shorten outer hair cell stereocilia, hindering the connection between the tectorial membrane and the tip of inner hair cell stereocilia. This changes the subtectorial geometry and increases endolymph flow, damaging inner hair cells, and explaining the progressive hearing loss. 16

In the present study, the TEOAE was absent in 8 (53.3%) out of the 15 subjects assessed and, among these subjects, 6 also presented hearing loss. The mean SNR in subjects in whom the TEOAE was present was significantly lower than in the CG, and the higher the frequency, the greater the difference between the groups ( Fig. 3 ).

The analysis of TEOAE amplitude only considered the results of the patients who presented TEOAE responses, so only 2 of the 7 who presented TEOAE had hearing loss, which was characterized by thresholds at 30 dB HL at 6 kHz and 8 kHz, which would not influence TEOAE responses.

It is also important to note that the protocol for recording TEOAEs used in the present study considered the frequencies of 1 kHz, 1.5 kHz, 2 kHz, 3 kHz, and 4 kHz, and a response in at least 3 frequencies was considered the presence of TEOAE. Considering that none of the patients presented middle ear impairment and that the sensorineural hearing loss observed in 8t patients affected frequencies above 3 kHz, the absence of TEOAE in the patients in the present study could not be explained by hearing loss, much less the lower in amplitude observed at the lower frequencies of 1 kHz, 1.5 kHz, and 2 kHz. Besides this, the absence or decreased amplitude of TEOAE responses was found even in the presence of hearing thresholds within normal limits, which demonstrates that individuals with WS present cochlear dysfunction, mainly in the basal regions of the cochlea.

These data corroborate the findings of other studies 7 8 11 14 which suggest subclinical changes related to the absence of OAEs in individuals with WS.

A study 12 found absent OAEs in subjects with hearing loss and normal hearing thresholds. According to the authors, 12 this finding may point to a failure in the medial olivocochlear efferent system, which is responsible for controlling mechanical outer hair cell movements. A failure in this system would lead to an irreproducible tracing, which would be interpreted as an absent TEOAE.

Some authors 7 researched distortion-product otoacoustic emissions and found a notch at 4 kHz; they correlated this finding to the absence of cochlear compression in 85% of the assessed subjects, and pointed out that this change could predispose subjects with WS to hearing losses related to high sound pressure levels.

Contrary to the aforementioned studies 12 13 the findings of the present one show that only 2 (25%) of the subjects with absent TEOAE presented normal hearing thresholds. Such a difference may have occurred because the present study assessed only adults, who already have perceptible auditory damage characterized by increased hearing thresholds at higher frequencies. Despite the many subjects assessed in the study by Fraga et al., ^12^ they were mainly children – as in the study by Fagundes Silva et al., ^13^ whose subjects mostly had normal hearing thresholds. These data reinforce the statement that subjects with WS may have progressive hearing loss, presenting OAE that indicates signs of subclinical changes in childhood, which are verified in adulthood with abnormal psychoacoustic thresholds.

The ABR result analysis showed abnormalities in at least one ear in all patients with WS, with increased latencies in at least one wave regarding absolute or interpeak interval latencies. The main abnormality found in the ABR was increased absolute latency in waves I (in the right ear in 53.3% of abnormal results and the left ear in 60% of them) and III (in the right ear in 73.3% of abnormal results and the left ear in 46.6% of them). When comparing latencies between groups, a longer wave III latency was observed in individuals with WS compared with the controls, and a trend toward statistical significance was observed for wave I. Despite this, the percentage of abnormalities in wave V and interpeak interval was small, whereas there were no differences between the groups for wave V latency or any of the interpeak intervals.

These results, in the absence of middle ear impairment, suggest an alteration in acoustic transmission in the synapse between the inner hair cells and the distal portion of the auditory nerve, causing a delay in the generation of wave I, in WS individuals. The delay in wave III latency, in turn, may be related to the delay in wave I, since the interpeak interval was not increased compared with the CG.

In the literature, increased latencies were found in waves I, III, and V in individuals with WS in comparison with individuals with typical development, though with normal interpeak interval values. According to the authors, 25 waves III and V were prolonged due to the increase in wave I latency, and they concluded that there was a desynchronization in auditory nerve fibers or dysfunction in the interaction between cochlear hair cells and the distal portion of the auditory nerve. Nonetheless, the authors 25 do not rule out subjects with middle ear impairments in the study group, which suggests that the latency delay in wave I they found can also be due to conductive changes present in this population.

The findings of the present study did not agree with those of other studies, 10 13 as the authors found ABR with normal latencies and interpeak intervals in waves I, III, and V, and concluded that retrocochlear abnormalities were absent in individuals with WS. Again, this difference may be correlated with the age of the subjects assessed in each study.

As other studies have already mentioned, hearing loss in subjects with WS is progressive and suggests cochlear weakness. Hence, ABR results in childhood may undergo significant changes over time. In the literature, there are no studies that performed longitudinally assessments of the ABR in subjects with WS – which would be an interesting approach to verify changes in absolute latencies, interpeak intervals, and amplitude of responses to this potential in all phases of the lives of these subjects

The results of the present study highlight the importance of including audiological assessments in the set of yearly examinations of individuals with WS. Furthermore, this population needs a specific assessment protocol that includes not only basic audiological assessments (with audiometry and acoustic immittance), but also OAE research to detect subclinical signs of hearing changes, monitor their hearing, and instruct their families about the risks of hearing loss, thus providing them better quality of life.

## Conclusion

Individuals with WS present early and progressive cochlear abnormalities that affect mainly the basal region of the cochlea. As for the central auditory nervous system, this population may present brainstem alterations affecting mainly the lower brainstem, which seem to begin in adulthood.
